# Fracture Resistance of CAD/CAM Nanohybrid Resin Occlusal Veneers Based on Bonding Surface: Enamel vs. Enamel-Dentin vs. Enamel-Resin Coating

**DOI:** 10.4317/jced.62793

**Published:** 2025-06-01

**Authors:** José Giancarlo Tozo-Burgos, César Fernando Juárez-Vizcarra, Roger Calla-Poma, Marco Sánchez-Tito

**Affiliations:** 1 Research Group on Dental Biomaterials and Natural Products, Faculty of Health Sciences, Universidad Privada de Tacna, 23000 Tacna, Peru; 2 School of Dentistry, Faculty of Health Sciences, Universidad Privada de Tacna, 23000 Tacna, Peru; 3 Faculty of Dentistry, Universidad Nacional Mayor de San Marcos, 15001 Lima, Peru

## Abstract

**Background:**

Occlusal veneers provide an effective alternative for treating severe tooth wear. The aim of this study was to evaluate the influence of the bonding surface on the fracture resistance of CAD/CAM nanohybrid resin occlusal veneers with different thicknesses.

**Material and Methods:**

A total of 62 occlusal veneers were fabricated for first premolars. The specimens were divided into two groups of 36 based on the thickness of the veneers, (1.0 mm and 1.5 mm). Each group was further subdivided according to the bonding surface, categorized as enamel, enamel-dentin, and enamel-resin coating. The fracture resistance of the veneers was evaluated by applying a constant load at a speed of 1 mm/min using a universal testing maching. After fracture of the samples, they were analyzed using a stereomicroscope. Student’s t-test was employed to compare fracture resistance in relation to thickness and bonding surface. Addionally, two-way ANOVA was used to assess the interaction between the variables. The significance level was set to 5%.

**Results:**

The 1.5 mm occlusal veneers exhibited significantly higher fracture resistance compared to the 1.0 mm veneers (*p*< 0.05). The two-way ANOVA test indicated that the interaction between restoration thickness and bonding surface was not significant (*p* = 0.3008). However, both restoration thickness (*p*< 0.001) and bonding surface (*p*< 0.001) had a significant impact on fracture resistance.

**Conclusions:**

Fracture resistance was highest in CAD/CAM nanohybrid resin occlusal veneers bonded to enamel and resin coatings. Likewise, restorations with a thickness of 1.5 mm showed significantly higher values across all three groups evaluated.

** Key words:**CAD/CAM, Occlusal veneers, Resin-coating technique, composite resin, fracture resistance.

## Introduction

Recent advancements in dental sciences have introduced new therapeutic strategies, optimizing clinical practice and improving the predictability of treatment outcomes (1-3). Current restorative procedures adopt biomimetic approaches, aiming to replicate the natural properties of teeth using minimally invasive techniques. This involves the use of materials with mechanical and optical characteristics that closely resemble those of dentin and enamel (4-8). In this context, occlusal veneers offer an effective restorative alternative, particularly for patients with significant tooth wear in the posterior region. Unlike full-coverage crowns, occlusal veneers help preserve a larger amount of dental tissue, reducing the risk of excessive wear on the underlying substrate (9,10).

Clinicians must understand the various tooth preparation designs and substrate conditioning for this type of restoration, as these factors significantly influence clinical outcomes such as fracture resistance and marginal adaptation (11). A minimally invasive tooth preparation approach, like anatomical occlusal reduction, offers greater fracture resistance compared to designs that incorporate rounded shoulders or central grooves (12). Additionally, studies have shown that when using CAD/CAM ceramic and resin occlusal veneers, substrates like dentin, composite, or immediate dentin sealing demonstrate the highest fracture resistance values (13,14).

Regarding the materials, occlusal veneers made with nanohybrid resin CAD-CAM blocks demonstrate superior fatigue performance compared to glass ceramics. These veneers also exhibit high fracture resistance, even in restorations with minimal thicknesses of 0.3 mm and 0.5 mm, while they can reach maximum thicknesses of 1.5 mm. This strength exceeds the average occlusal force values found in the posterior dentition, which typically range from 585 to 880 Newtons (15-19). Additionally, a noTable advantage of these materials is that they do not cause any alterations to the surface of the opposing tooth (20,21).

Regarding their durability in the mouth, a controlled clinical trial with a three-year follow-up period demonstrated similar durability between ultra-thin glass-ceramic occlusal veneers and nanoceramic resins in treating severe dental erosion. The only significant difference observed was in surface roughness, as the resin group experienced some surface degradation (22). This indicates that these restorations are a viable option for patients with bruxism. Evidence suggests that forces greater than 1000 N are required to cause cracks in restorations made with hybrid CAD/CAM resins that are 1.6 mm and 2.0 mm thick (23).

The dental market currently provides various restorative options for treating severe tooth wear. In many cases, specific clinical situations necessitate prior treatments on the dental surface to optimize the conditions for the substrate. However, the selection of restorative material is often primarily based on its inherent characteristics. Evidence suggests that it is also crucial to consider the bonding surface where the restoration will be cemented, as well as the appropriate thickness based on the clinical situation.

Therefore, the aim of this study was to evaluate the influence of the bonding surface on the fracture resistance of CAD/CAM nanohybrid resin occlusal veneers with different thicknesses.

## Material and Methods

- Study design and sample size calculation.

This *in vitro* experimental study received approval from the Ethics Committee of the Faculty of Health Sciences at Universidad Privada de Tacna, with registration number FACSA-CEI/59-03-2024.

The sample size calculation was conducted using G*Power version 3.1.3 (Heinrich Heine Universität, Düsseldorf, Germany). The calculation considered a two-way ANOVA test with interaction, an effect size of 0.4034, a significance level (α) of 0.05, a statistical power of 0.8, five degrees of freedom, and a total of six groups.

The study involved a sample size of 72 specimens, which were divided into two groups of 36 specimens each, based on the thickness of the occlusal veneers (1.0 mm and 1.5 mm). Each group was further subdivided into three subgroups according to the type of bonding surface: enamel, enamel-dentin, and enamel-resin.

- Sample preparation

The samples included occlusal veneers made from CAD/CAM nanohybrid resin (Evolux Hybrid; BlueDent, Alium Comercial), a recently introduced material in the market. These veneers were bonded to upper first premolars that had no caries or previous restorations and displayed similar dimensions in height, mesiodistal width, and buccolingual width. They were obtained through voluntary donations, with informed consent, and were extracted for orthodontic reasons. The teeth were stored in a 0.1% thymol solution until they were used. Each tooth was secured in a 3 cm high polyvinyl chloride tube (3/4 inch) and aligned along its longitudinal axis using self-curing acrylic resin. The cement-enamel junction was left exposed 2 mm above the level of the acrylic resin.

The occlusal surface was prepared with a 150° angle between the cusps, following a standard anatomical sequence for occlusal veneers. An 893H diamond bur (Jota AG, Rüthi, Switzerland) was used until the three types of bonding surfaces defined in this study were achieved. These criteria were established based on previous research (13,24).

**Figure 1:**
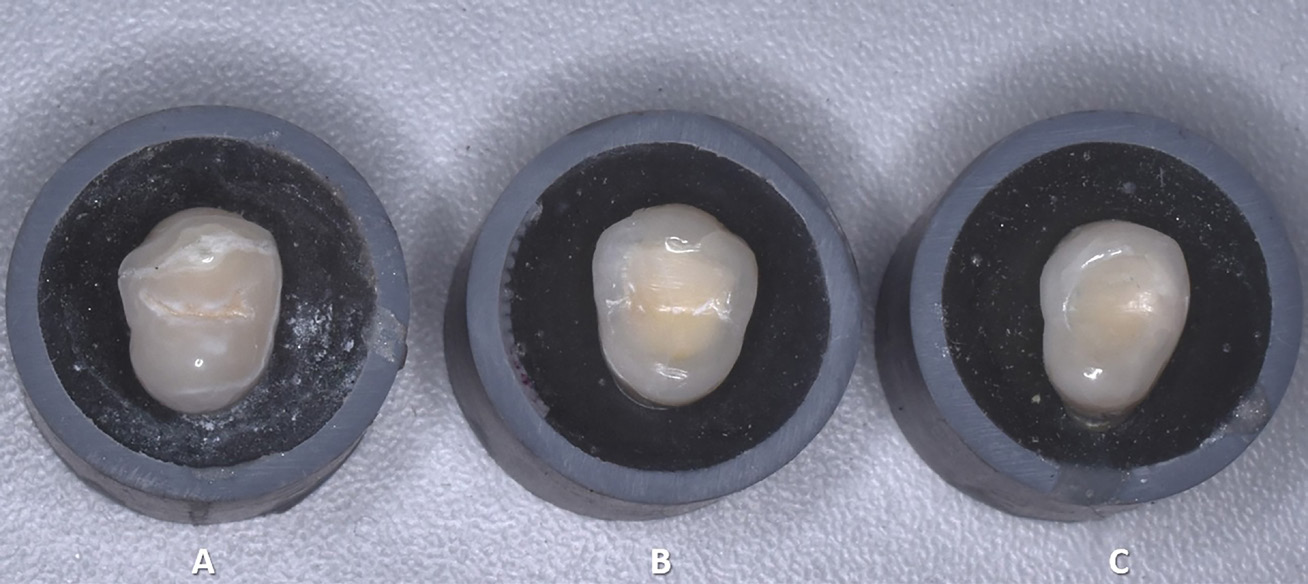
Dental preparation for occlusal veneers. A: Enamel only, B: Enamel-dentin, C: Enamel-resin coating.

In the first group, the preparation limited exclusively on the enamel. In the second group, the preparation extended into the dentin while leaving the surrounding enamel intact. In the third group, the resin coating procedure was applied in addition to the dentin preparation, resulting in uncovered peripheral enamel and the dentin being covered with resin (Fig. 1).

For the Resin Coating procedure, All-Bond Universal Adhesive (BISCO Inc., Schaumburg, IL, USA) was applied in two active layers on the exposed dentin, with each layer rubbed in for 15 s. After application, the solvent was evaporated using air bursts for 15 s. This was followed by photopolymerization using a Bluephase N lamp (Ivoclar, Schaan, Liechtenstein) set at 1200 mW/cm² for 20 s. Next, a 0.5 mm layer of Filtek Supreme Flowable Restorative (3M, St. Paul, MN, USA) was spread evenly across the entire dentin surface, which was then photopolymerized for another 20 s. Finally, a layer of glycerin was applied on top of the resin, followed by a final photopolymerization at the same intensity and duration (25-27).

- Fabrication of occlusal veneers

**Figure 2:**
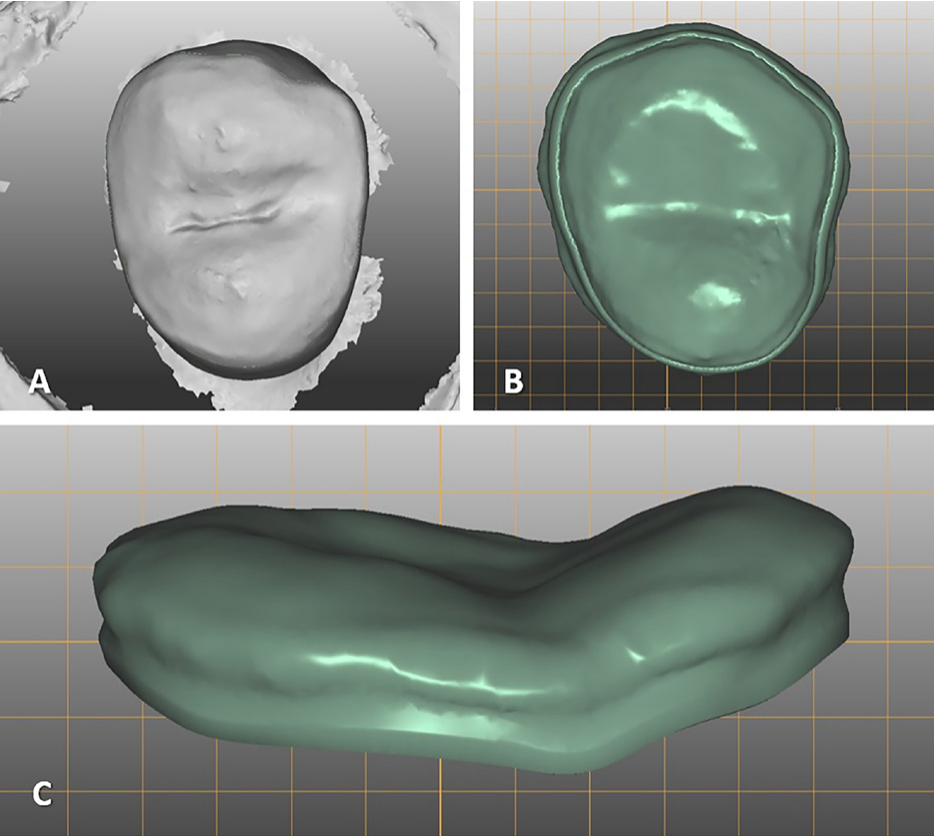
A: Digitization of the prepared tooth, B: Occlusal veneer design (occlusal view), C: Occlusal veneer design (lateral view).

The Arti scanner (S600, Zirkonzahn, Gais, Italy) was utilized to digitally capture each prepared tooth. Following this, occlusal veneers were designed using Zirkonzahn-Modellier software (Zirkonzahn, Gais, Italy) and produced with uniform thicknesses of 1.0 mm and 1.5 mm (Fig. 2).

The Compact Line Milling Unit M1 (Zirkonzahn, Gais, Italy) was utilized for the fabrication of the restorations. After the milling process, the restorations were polished using the Sof-Lex™ Diamond Polishing System (3M ESPE, St. Paul, MN, USA). The polishing was carried out in several steps: first, rough grinding was performed with the Sof-Lex™ Pre-polish Spiral discs. Next, the fine-grained Sof-Lex™ Finishing Spiral disc was used. Finally, diamond paste was applied in conjunction with brushes that were impregnated with abrasive particles. This process resulted in occlusal veneers with the specified thicknesses required for the study.

- Conditioning of bonding surfaces

For enamel and enamel-dentin substrates, Ultra-Etch 35% Phosphoric Acid (Ultradent Products Inc., South Jordan, UT, USA) was applied to the enamel for 20 s and then rinsed thoroughly with water for 30 s. After rinsing, the enamel surface was completely dried, while the dentin was kept slightly moist without being dried out. The All-Bond Universal Adhesive was then applied passively to the enamel and actively to the dentin by rubbing for 15 s. Following this application, the solvent was evaporated using air bursts for 15 s. Finally, light curing was performed for 20 s with a Bluephase N lamp, which has a power output of 1200 mW/cm² (28).

For the enamel-Resin coating substrate, sandblasting with 50 µm aluminum oxide was performed on the entire resin surface using a Standard Microblaster (Bio-Art, São Paulo, Brazil) for 10 s at a pressure of 1–1.5 bar (29). Following this, the surface was rinsed with plenty of water, and Ultra-Etch 35% Phosphoric Acid was applied to the enamel for 20 s. After washing and drying the surface, All-Bond Universal Adhesive was applied. The solvent was then dissipated using air bursts for 15 s, and photopolymerization was carried out for 20 s with a Bluephase N lamp, which has a power of 1200 mW/cm².

- Conditioning of the internal surface of occlusal veneers

Air abrasive blasting was conducted using 100 µm aluminum oxide with the Basic Quattro IS sandblaster (Renfert, Hilzingen, Germany) at a pressure of 2.5 bar for 15 s. The restorations were subsequently cleaned in an ultrasonic unit (Codyson, Shenzhen, China) with 70% ethanol for 5 min. They were then rinsed with water spray and gently dried using air bursts. Finally, a thin layer of All-Bond Universal Adhesive was applied, and the solvent was evaporated with air before being light-cured for 20 s using the Bluephase N lamp at a power output of 1200 mW/cm² (14).

- Cementation of occlusal veneers

Allcem Veneer APS resin cement (FGM Dental Group, Joinville, Brazil) was applied to the entire restoration before it was placed on the prepared tooth using firm pressure. Any excess cement was removed with a brush, and then photopolymerization was carried out using a Bluephase N lamp at a power of 1200 mW/cm² for 20 s on each surface (occlusal, vestibular, lingual, and proximal). The margins were polished 24 h later using abrasive rubbers (14).

- Fracture resistance test

**Figure 3:**
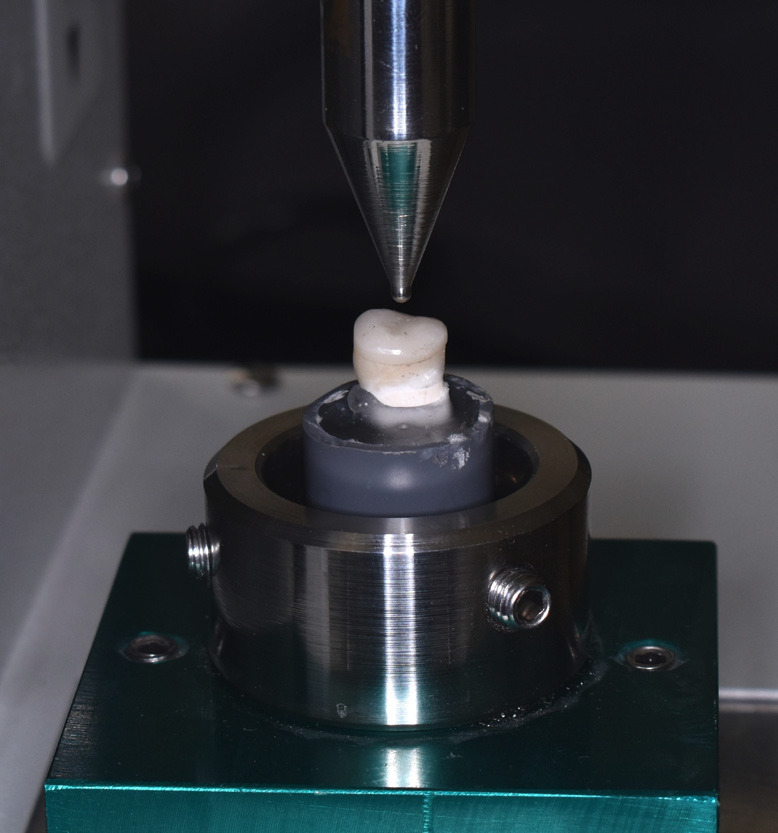
Fracture resistance test on a universal testing machine.

A universal testing machine (OM 150, Odeme Dental Research; Joacaba, SC, Brazil) equipped with a 2.5 mm diameter spherical actuator was used to assess the fracture resistance of the samples. The load was applied vertically to the central fossa of the restoration at a speed of 1 mm/min until fracture occurred. The recorded fracture resistance values were measured in Newtons (N) (15) (Fig. 3).

After conducting the fracture resistance test, all samples were examined under a Trinocular Stereomicroscope at a magnification of 20x (AmScope SM20, United Scope LLC, USA) to identify the type of failure. The classification system used was based on the criteria established by Saleh *et al*. (30), which includes type 1: restoration fracture; type 2: combined restoration and tooth fracture; and type 3: root fracture.

- Statistical analysis

Statistical analysis was conducted using Stata® software version 17 (StataCorp LP, College Station, TX, USA). The Shapiro-Wilk test was performed to evaluate the normal distribution of the data, while the Breusch-Pagan/Cook-Weisberg post-estimation test was utilized to assess homoscedasticity. Student’s t-test was employed to compare the fracture resistance of the veneers based on the thickness of the restoration and the bonding surface. Additionally, a two-way ANOVA test analyzed the impact of restoration thickness and bonding surface on the fracture resistance of occlusal veneers, including any interaction effects. Pairwise comparisons were made using marginal values calculated with the Bonferroni method. The significance level was set at *p* < 0.05.

## Results

 Table 1 shows the comparison of fracture resistance in occlusal veneers based on thickness and bonding surface. The results indicate that for all three bonding surfaces, veneers that are 1.5 mm thick demonstrate greater fracture resistance compared to those that are 1 mm thick. This difference was statistically significant (*p* < 0.05).

The two-way ANOVA test indicated that the interaction between restoration thickness and bonding surface did not have a significant effect on fracture resistance (F = 1.22; *p* = 0.3008). This model accounts for 84.9% of the observed variability in the fracture resistance values of the occlusal veneers (ηp2 = 0.849). Additionally, the results demonstrate that both the main effect of restoration surface thickness (F = 51.55; *p* < 0.0001) and the main effect of bonding surface (F = 113.92; *p* < 0.001) significantly influence the fracture resistance of the nanohybrid resin occlusal veneers ( Table 2).

 Table 3 presents the results of multiple comparisons between groups based on restoration thickness and bonding surface. All comparisons among the various combinations showed statistically significant differences (*p* < 0.001), except for the following cases: the comparison between the 1 mm thickness with enamel only and the 1.5 mm thickness with enamel only (*p* = 0.056), the comparison between the 1 mm thickness with enamel-dentin bonding and the 1.5 mm thickness with enamel-dentin bonding (*p* = 1.000), and the comparison between the 1 mm thickness with enamel-resin coating bonding and the 1.5 mm thickness with enamel-dentin bonding (*p* = 1.000).

**Figure 4:**
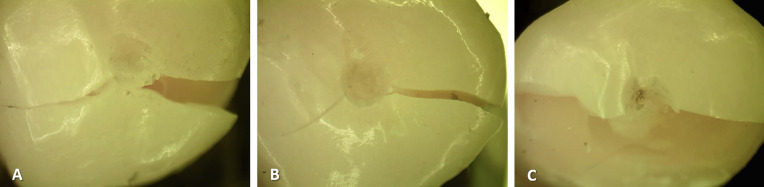
Evaluation of the type of failure according to the adhesion surface with a stereomicroscope. A: Enamel only (Type 1), B: Enamel-dentin (Type 1), C: Enamel-resin coating (Type 1).

Finally, the stereomicroscope evaluation revealed that in all samples, the type of failure occurred at the level of the restoration, aligning with type 1 of Saleh *et al*.’s classification (Fig. 4).

## Discussion

This study assessed how the bonding surface influences the fracture resistance of CAD/CAM nanohybrid resin occlusal veneers with thicknesses of 1 mm and 1.5 mm. The results showed statistically significant differences in fracture resistance related to both the bonding surface and the thickness of the restoration.

The present study found that increasing the thickness of CAD/CAM nanohybrid resin occlusal veneers significantly improved their fracture resistance (*p*<0.05). This enhancement was consistent across all analyzed bonding surfaces. These results are in agreement with those of Czechowski *et al*. (31) and Ioannidis *et al*. (32), who reported that thicker occlusal veneers demonstrate greater fracture resistance, regardless of the material used. Additionally, their studies, which examined nanoceramic resins (Lava Ultimate) and polymer-infiltrated ceramics (Vita Enamic), highlighted that a greater thickness of restoration improves stress distribution and reduces the likelihood of internal defects, thereby enhancing fracture resistance.

In this study, occlusal veneers bonded to enamel/resin coating exhibited the highest fracture resistance values. These results are consistent with those reported by Sasse *et al*. (13), who examined the influence of three different substrates on the fracture resistance of occlusal veneers and found that bonding to enamel/resin was the most effective. However, their study utilized lithium disilicate as the selected material. The similarity between their findings and ours suggests that enamel/resin coating adhesion may improve the mechanical performance of polymeric and glass-ceramic materials.

Few studies have analyzed the behavior of CAD/CAM resins about the three bonding surfaces evaluated in this research. One notable study is by Mueller *et al*. (14), which examined the effect of immediate dentin sealing (IDS) on the mechanical performance of CAD/CAM occlusal veneers made of glass-ceramic and composite resin. Their findings demonstrated that IDS significantly improves the accelerated fatigue resistance of CAD/CAM composite resin occlusal veneers compared to those cemented directly to dentin without IDS. In our study, we focused on a specific type of bonding surface and applied the Resin Coating technique, which is a variation of the IDS method. The main distinction between these two techniques lies in the materials used: Resin Coating employs self-etch adhesives, while IDS uses etch-and-rinse adhesives, which require prior acid etching of the tooth surface. Despite this difference in materials, both techniques create a hydrophobic resin layer, leading to similar surface properties. This similarity may explain the superior mechanical performance observed in occlusal veneers bonded to this type of substrate, particularly those made from resin composite materials. This approach enhances adhesive bond strength, optimizes stress distribution, and improves the absorption of masticatory forces. As a result, it leads to better marginal adaptation and promotes more effective chemical integration between the cementing resin and the restoration (25-27). These benefits are likely to be more pronounced in polymeric materials than in glass-based materials, owing to the former’s superior chemical compatibility and their enhanced ability to distribute stresses generated during masticatory function (33,34).

The lowest values for fracture resistance were found in occlusal veneers bonded to enamel. This finding contradicts some fundamental concepts of dental adhesion, which suggest that enamel is the ideal substrate for adhesive procedures due to its high inorganic hydroxyapatite content (96%) and the perpendicular arrangement of its prisms. These characteristics are thought to reduce stress absorption compared to dentin, which should theoretically enhance the mechanical properties of restorations bonded to this substrate (35-37). However, enamel exhibits greater biomechanical compatibility with glassy ceramic materials, such as lithium disilicate, because their mechanical properties are similar to those of enamel (38). Gierthmuehlen *et al*. (39) investigated how the thickness of ceramic occlusal veneers and the type of dental substrate affect their survival rate and the load required for mechanical failure. Their findings indicated that occlusal veneers bonded to 0.5 mm of enamel demonstrated superior mechanical performance compared to those that were 1.0 mm thick. This suggests that minimal thicknesses of lithium disilicate veneers enhance mechanical resistance, especially when applied to highly mineralized substrates. These results could help explain the variability observed in our study, as the occlusal veneers we used were made from a material with different mechanical properties than lithium disilicate.

Regarding the material used, the Evolux Hybrid CAD/CAM nanohybrid resin has been recently introduced to the market, and most of the available information comes directly from the manufacturer. In our study, this CAD/CAM resin exhibited fracture resistance values ranging from 768.13 ± 24.44 N to 915.28 ± 24.58 N, which are considered adequate for a definitive restoration. However, when compared to similar materials such as Lava Ultimate and PICN Vita Enamic, previous studies with comparable methodologies have shown that these materials achieve fracture resistance values exceeding 1200 N, even at minimal thicknesses of 0.5 mm (19,23,32). This difference may be attributed to variations in the composition of each material. Specifically, PICN Vita Enamic consists of 75% feldspathic ceramic and 25% polymer, whereas Lava Ultimate contains 80% ceramic nanoparticles (40). In contrast, the manufacturer reports that Evolux Hybrid is composed of 40-45% ceramic nanoparticles and 55-60% polymer by volume (41).

Despite these differences, the fracture resistance values of Evolux Hybrid exceed the minimum threshold for definitive restorations. This is particularly relevant, as the bite force in the posterior region of healthy adults ranges from 300 to 630 N, while in individuals with parafunctional habits, such as bruxism, it can reach between 880 and 1000 N (42-45). These findings suggest that the selection of this material should be assessed based on the specific clinical needs of each patient.

One of the main limitations of this study is its *in vitro* nature, which does not completely replicate the conditions of the oral environment. Furthermore, there is currently a lack of studies that analyze the mechanical behavior of CAD/CAM resins in relation to the bonding surface. Additionally, there is not enough detailed information available about the materials used in this research.

Future studies are recommended to evaluate the effect of the cementing agent on the mechanical performance of CAD/CAM nanohybrid resins, taking into account the substrate that demonstrated the best results in this study.

## Conclusions

Fracture resistance was highest in CAD/CAM nanohybrid resin occlusal veneers bonded to enamel/resin coating. Similarly, restorations with a thickness of 1.5 mm exhibited significantly higher values across all three groups evaluated.

## Figures and Tables

**Table 1:** Means and standard deviations for fracture resistance (Newtons).

Restoration thickness	Bonding surface		
	Enamel	Enamel/dentin	Enamel/resin coating
1.0 mm	768.13 ± 24.44^a^	810.39 ± 26.63^a^	871.88 ± 25.67^a^
1.5 mm	799.09 ± 23.76^b^	864.12 ± 26.27^b^	915.28 ± 24.58^b^

Note: Different lower letter in the columns indicates statistically significant differences. Student’s t test for independent samples.

**Table 2:** Two-way ANOVA for fracture resistance main effects and interactions between restoration thickness and bonding surface.

Source	Partial sum of squares	df	Mean square	F	Sig.	Partial Eta squared
Model	179724.97*	5	35944.99	56.37	<0.001	0.849
f1	32870.907	1	32870.907	51.55	<0.001	0.568
f2	145293.78	2	72646.891	113.92	<0.001	0.828
f1*f2	1560.284	2	780.14202	1.22	0.3008	0.137

Note: f1: Restoration thickness (main effect), f2: bonding surface (main effect), and interactions. Abbreviation: df, degree of freedom. * R-squared = 0.810 (adjusted R2 = 0.7959).

**Table 3:** Pairwise comparisons of marginal linear predictions (Newtons).

Restoration Thickness	Bonding surface	Fracture resistance
1.0 mm	Enamel	768.13 ± 24.44^a^
	Enamel/dentin	810.39 ± 26.63^b^
	Enamel/resin coating	871.88 ± 25.67^c^
1.5 mm	Enamel	799.09 ± 23.76^ab^
	Enamel/dentin	864.12 ± 26.27^c^
	Enamel/resin coating	915.28 ± 24.58

Note: there is no difference between the same lower letter in the column (interaction of restoration thickness x bonding surface). Bonferroni method for pair-wise comparison. The level of significance was set at *p* < 0.003.

## Data Availability

The datasets used and/or analyzed during the current study are available from the corresponding author.
